# Public health systems analysis - where the River Kabul meets the River Indus

**DOI:** 10.1186/1744-8603-9-39

**Published:** 2013-08-30

**Authors:** Colin P Thunhurst

**Affiliations:** 1 Honorary Principal Research Fellow Faculty of Health and Life Sciences Coventry University, 5 Aire View Sandbeds, BD20 5LH Keighley, West Yorkshire, UK

**Keywords:** Public health, Systems analysis, Operational research, Reverse innovation

## Abstract

In this paper we review two recent paradigmatic shifts and consider how a two-way flow in innovation has been critical to the emergence of new thinking and new practices. The first area relates to our understanding of the nature of public health systems and the shift from a medical paradigm to a more holistic paradigm which emphasises the social, economic and environmental origins of ill-health and looks to these as key arenas in which to tackle persistent inequalities in populations’ health experiences. In respect of this paradigmatic shift, it is argued, developing countries were in advance of their more developed counterparts. Specifically, the Alma Ata Declaration and the *Primary Health Care Approach* which was central to its implementation pre-figured elements of what was to be called in developed countries *The New Public Health* such as the need for greater community involvement and recognition of the importance of other sectors in determining health outcomes. But this paradigmatic shift added a new complexity to our understanding which made the identification of appropriate policy responses increasingly difficult. However, a parallel shift was taking place in the cognate field of operational research/systems analysis (OR/SA) which was adding greatly to our ability to analyse and to identify key points of intervention in complex systems. This led to the emergence of new techniques for problem structuring which overcame many of the limitations of formal mathematical models which characterised the old paradigm. In this paradigmatic shift developed countries have led the way, specifically in the new fields of Community Operational Research and Operational Research for Development, but only by drawing strongly on the experience and philosophies to be found in developing countries.

## Background

When driving the Grand Trunk Road from Rawalpindi to Peshawar it is always a good idea to take a cup of the local green tea at the chai-stop at Attock. This is the border between the Punjab and the Khyber Pakhtunkhwa Provinces of Pakistan. (Khyber Pakhtunkhwa is the new official name for the North West Frontier Province and will be referred to by the more familiar acronym NWFP below). It is at this point that you cross the River Indus. But it is also the point at which the River Kabul joins the River Indus. The Kabul, having come from Afghanistan and crossed the sandy planes of NWFP, is browny-red. The Indus, having descended from the melting glaciers of the Himalayas, is a clear blue. After a few hundred metres they combine into a single flow; but for a brief while the respective rivers hold to their distinct characteristics. The confluence provides a perfect metaphor for the paradigm shift currently underpinning our changing understanding of public health systems.

Like the rivers at the confluence *Public Health Systems Analysis* is an emergent meta-discipline which bears hall-marks of its respective antecedents. These antecedents are the *Whole Systems Thinking* which is currently driving our understanding of public health systems and the *Whole Systems Analysis* which has emerged from disciplines such as Operational Research and which is adding an analytic rigour to the change in conceptual thinking. As we will describe below, both currents have flourished as a consequence of global perspectives moulded in both developed and developing countries.

In this paper we look first at the development of whole systems thinking in relation to public health systems noting how it has been reflected in the model of comprehensive primary health care for developing countries that was seen as achieving the aspirations of the Alma Ata Declaration and in the model of the New Public Health Movement that emerged subsequently within the developed world. This is followed by a consideration of the development of complimentary ‘new paradigm’ techniques of analysis known as problem structuring methods that have been driven (amongst other things) by the analytical requirements of two closely-related fields of systems analysis, *community operational research* and *operational research for development*, both of which have spanned the developing and the developed worlds. We then look in more detail at some examples taken from both developed and developing world contexts of where these analytical techniques have been used to enhance the planning process for public health systems interventions. We close with some observations on the implications of the respective fusions, between whole systems thinking and whole systems analysis and between the practical experiences of developed and developing countries, on public health planning and public health practice across the globe.

### Public health whole systems thinking

Health policy analysts from the developing countries were ahead of their counterparts from the more developed world in bringing holistic thinking to their understanding of public health systems. The Alma Ata Declaration of 1978 [1] was initially a response born of necessity. Developing countries were increasingly realising that the urban-focussed curative-oriented health systems that they had inherited from colonialism were failing, in a very expensive way, to meet the health care needs of their majority populations. The model of Primary Health Care [2] that followed was aimed at addressing the underlying determinants of ill health (root causes) rather than simply focussing on remedial measures to tackle the resultant ill health (consequences). This shift from a medical model to a more holistic health-oriented model had important implications for health planning. As well as orienting health care delivery towards preventive and health promoting interventions, the model highlighted the important contribution of non-health sectors. Inter-sectoral collaboration and the cross-sectoral interventions that the model aspired to promote were seen to be equal in importance for improving health status as were the clinical interventions that were growing increasingly out of the economic reach of developing countries.

The developed countries came to a more gradual realisation of the need to re-think their approach to seeking health improvement. If there was a single driving force it was the belated rediscovery of the existence and of the persistence of health inequality. The United Kingdom, which had been in the forefront of developing a socialised health care delivery system, typified this ‘sudden awakening’. The Black Working Party, (so-called because it was chaired by Sir Douglas Black), was commissioned by a soon-to-be-outgoing Labour Government to investigate the extent to which the UK National Health Service was delivering equality of health opportunity. When the working party reported [3], in 1980, a new government had come into office carrying an ideological perspective to which the major recommendations of the working party were anathema. Despite their best efforts to ‘shelve’ it (and to some extent because of them) the contents of the report were widely circulated. Even to sympathetic commentators the findings were eye-opening. Despite growing discussion of ‘diseases of affluence’ it was seen that with remarkable consistency the health hazards of advanced economies were distributed in an inverse relationship to the material fruits of those economies. The most extreme examples of this inverse relationship, such as road traffic accidents, came from areas where remedial measures were most clearly outside of the realm of conventional health care delivery systems.

Within developed nations there was talk of a *New Public Health*[4]. Though, as was readily acknowledged by the movement’s founders, the newness owed much to a re-appreciation of the more intuitively driven public health initiatives undertaken by their predecessors in the Victorian period over 100 years earlier. {At that time public health officers had not waited for a detailed understanding of the causes of tuberculosis, for example, to appreciate the contribution of social factors such as housing and nutrition in addressing the causes of this then major killer}. The New Public Health movement, drawing upon this tradition and echoing the primary health care approach being increasingly pursued within developing countries, argued for a health policy built on an appreciation of the need to adopt a more holistic vision – a vision which acknowledged the contribution of a wide range of environmental, social and economic forces in shaping the production and distribution of ill health.

In common with paradigmatic shifts in all scientific and social scientific fields, the emergence of new paradigmatic thinking has met with resistance from the old paradigm. Within public health, the outgoing paradigm is the medical model. As a consequence, and notwithstanding the gradual (and sometimes reluctant) acceptance of the new vision, translation into policy has progressed more slowly than might have been hoped, in both developed and developing nations. Within the developing world, with the active encouragement of donor agencies conscious that greater (and more widely spread) short term social investment might be needed to achieve longer term health gain, policy analysts introduced the concept of *selective primary health care*[5]. Whilst paying lip-service to the principles of the underlying integrated model this approach argued for the progressive ‘picking off’ of major health problems. Buoyed by the successful world-wide drive to eradicate smallpox, this approach has led to a proliferation of vertical programmes. Typically, there will be separately managed and funded programmes to address priority diseases such as malaria and tuberculosis and to deliver an Expanded Programme of Immunisation. Such programmes now pepper developing health systems. Notwithstanding recognition of their individual effectiveness (such as the much-lauded Lady Health Worker Programme in Pakistan) they stand as a heretical challenge to the integrated (or *comprehensive*) primary health care model as initially formulated in the wake of the Alma Ata Declaration and frequently represent wasteful duplication in the use of scarce health sector resources.

The globalisation of the common understanding that links the Primary Health Care Approach to the New Public Health Movement has been supported by the World Health Organisation. WHO regional offices have played a significant role in promoting this new understanding and adapting it to fit the circumstance of distinct world regions and of the nations within them. At the global level, the WHO’s Commission on the Social Determinants of Health [6] (chaired by Professor Sir Michael Marmot) has drawn attention to the overarching symmetry in understanding and the common threads of policy development between developed and developing nations. Again, we see the former taking its lead from the latter. In the wake of the global report The Marmot Strategic Review of Health Inequalities in England Post-2010 [7] once again reminded health sector decision-makers within a developed nation of the underlying factors producing and replicating health inequality and indicated the global commonality of the major policy drivers.

Within the developed world, it would appear to be political lethargy, underpinned by the tendency to build discrete organisational silos, (latterly enforced by economic retrenchment), which has held back the operationalisation of this more holistic vision. Political drift towards the right has not helped. Ideologically, both right and new left politicians have resisted the implied relationship between state and private enterprise that the New Public Health model calls for.

The intellectual impetus has become increasingly compelling, but it has also become increasingly complex. This has not been just the product of the unfortunate academic tendency to complexify understanding, but a genuine reflection of the increasing complexity of the globalised world. In drawing together their various recommendations aimed at reducing health inequalities within the UK, the Marmot Review team highlighted the need to adopt what it called a *whole*-*system approach*:

“Strategies that rely on intervention in one part of the system will be insufficient to make the necessary difference to patterns of inequality. A whole-system approach is needed in which organisation and people work together with activity at national, regional, local and individual levels” [7].

Within one specific area of policy, the relevance of this conclusion had previously been demonstrated by the work of a Foresight Committee established to investigate the underlying causes of the current ‘obesity epidemic’ [8]. The Committee identified a broad range of factors influencing obesity and explored the complex interactions between them. The initial report of the committee contained one figure that came to be known as the ‘spaghetti diagram’ in which the representation was so complex and detailed that it was impossible to pick out any individual elements with the naked eye (magnification to at least 200% was necessary). Further work by the Committee, which we will return to below, illustrated that thinking at the whole systems level requires accompaniment by appropriate methods of analysis at that level.

Also pre-empting the recommendations of the Marmot Review, a NICE (National Institute for Health and Clinical Excellence) scoping study on “preventing obesity using a *whole*-*system approach* at local and community level” provided some clarification on the implications of adopting such an approach.

“For the purpose of this guidance, a ‘whole-system’, sustainable approach to obesity involves a broad set of integrated policies combined with population-wide and targeted measures. This includes action by central and local government, industry, communities, families and society as a whole. It also involves shifting attention away from individual risk factors or isolated interventions and considering many influences simultaneously…” [9].

This valuable clarification (which might have been written in relation to any part of the world) also highlighted a further feature or a natural consequence of adopting *whole systems thinking* that was also to be explicitly addressed further by the Marmot Review. This is its relationship to community empowerment. The Marmot Review’s second recommendation was titled *Empowering people*: *securing community solutions* and called for “community engagement practices to move beyond what are often routine, brief consultations, to involving individuals in partnerships to define problems and develop local solutions to address those problems” [7]. As we will see below, this is the feature of whole systems thinking wherein the developed countries have perhaps the strongest need to learn from their less developed partners.

{In a recent text, Rayner and Lang [9] offer an “ecological” perspective on public health which provides a complimentary understanding pitched at a higher level of analysis, rather than the problem specific level of say the Foresight Committee. They identify a series of “transitions” which “collectively shape the population’s health and how people live their lives” which frame or provide the focus for public health interventions}.

### Whole systems analysis

The shift to Whole Systems Thinking – be it through the evolution of the Primary Health Care Model in developing nations or the emergence of the New Public Health within the developed world – constitutes a change in the way that we think about the achievement of health gain that constitutes a paradigmatic shift. Similar paradigmatic shifts were taking place in parallel disciplines, often of a nature that made them complementary or essential to achieving the potential of the new thinking on health systems.

Systems Analysis is a generic term that is currently used to encompass methods and approaches previously more commonly referred to as operational research (in the UK) or operations research (in the US). {In the US it has become the norm to employ the conjoint acronym OR/SA; in the UK OR/MS has been used to signify the relationship to the wider management sciences}. Operational research, as the name hints, started life as the application of the methods of science to military operations. In essence this meant deploying a range of established and newly developed modelling techniques – simplified representations of complex problem situations - which permitted experimentation and interrogation producing a fuller understanding which could then be transferred back to the ‘real life’ of complexity. The first major advances took place during the Second World War. But following the end of the war it was seen that many of the logistical and tactical issues facing private industries and, in the UK, the new nationalised industries and services, were similar in nature to operational problems in military planning. The methods, and many of the personnel, made a comparatively rapid shift to the civilian arena.

However, over time there was a growing dissatisfaction from within the professions at the limited range of problems that existing modelling techniques were able to analyse. These modelling techniques were predominantly mathematical in nature, requiring reliable quantitative data and a well-defined problem context, conditions which generally only applied within relatively short-term tactical or operational problem situations. As analysts turned their attentions to the more ‘messy’ world of strategic decision making these underlying problem characteristics were seen to be missing. A paradigmatic shift was necessary. {In classic Kuhnian terminology, the science of operational research was in crisis when confronted with this new set of problems}.

A new paradigm of operational research emerged around a set of techniques now grouped under the label *problem structuring methods* (PSMs) [10]. These techniques focussed more on the process of problem identification and exploration than they did on problem solution, reflecting a realisation that once a messy problem had been clearly defined the solution was often quite trivial. The techniques were less demanding of data, employing methods of qualitative modelling. The techniques were also more inclusive, as they did not accept the starting point of a centrally defined single problem definition but encouraged participation in the exploration of poorly defined problem spaces.

Some of the new paradigm techniques were adaptations of established modelling approaches – games theory for example evolved to give drama theory and confrontation analysis. Others of these techniques drew upon existing techniques from parallel disciplines. Strategic Options Development and Analysis (SODA) built upon the use of mapping in cognitive psychology; Soft Systems Methodology took forward the work of Peter Checkland. Others were developed ab initio – the strategic choice approach developed initially to support the work of the Tavistock Institute for Human Relations [10].

Two areas of application were critical to highlighting this need for a paradigm shift and to advancing the development and application of the newly emerging techniques. These were *Community Operational Research* and *Operational Research for Development*. As with the evolution of whole systems thinking in respect of the health sector these arenas for the development of whole systems analysis drew upon roots that were located in both developed and developing countries and, for their growth, required extensive cross-fertilisation.

### Community operational research

The importance of analysing health determinants at the community level was, for the Marmot Review, a natural (indeed essential) consequence of adopting a whole-system approach. As already mentioned, the review’s second set of recommendations addressed the critical importance of ‘Empowering people: securing community solutions’ [7].

The emergence of the community as an explicit arena for the application of OR/SA methods stemmed from growing awareness of the narrow field of application dominant in the period following the Second World War, up to the 1970s. The utilisation of formal mathematical models constrained their application to well-structured problems of a nature that could generally only be found within the larger more bureaucratic organisations. These predominantly came from the private and nationalised industries, though a limited range of applications had been made within the social services. What had been significantly missing had been applications within the community sector.

The limited field of application was largely (though not entirely) accounted for by the narrow range of modelling techniques deployed by OR/SA practitioners. Community organisations exhibit features and operate within contexts which can be classically characterised as ‘messy’ in nature. Decision-making involves dialogue and debate and objectives are either not well-specified, are contested or are multi-dimensional. Concepts such as ‘optimality’, which lie at the heart of most formal mathematical models, have only limited relevance in such problem contexts.

During the 1970s and 1980s the new field of Community Operational Research within the UK developed an impressive catalogue of work (see, for example Ritchie, Taket and Bryant, [11]). This would not have been possible without the paradigm shift which saw the emergence of the newly developed problem structuring methods; though, as some practitioners were keen to point out [12], even the traditional OR/SA methods did have their occasional moment.

Community Operational Research was a cross-cultural cross-continental movement. The Community Operational Research Unit, established in the UK, acknowledged its debt to the working philosophy of action research as articulated by the Society for Participatory Research in Asia [13]. Vidal [14] drew the parallel with the alternative consulting work of the Centro Latinamericano do Trabajo Social (CELAT) and other organisations in Latin America working in the traditions of Paolo Freire. At the methodological level, Thunhurst [15] advocated cooption of problem-solving tools such as “Mawas Diri” [16].

### Operational research for development

Taket and White [17] drew particular attention to the range of participatory appraisal methods developed within international development practice by the participatory movement closely associated with the work of Robert Chambers. There was, from the outset, a strong inter-relationship in terms of philosophical and methodological approach and even in terms of personnel between Community Operational Research and a parallel movement within OR/SA to advance Operational Research for Development.

This inter-relationship was built on recognition of the shared contexts of the community setting and the broader development setting (within which the community setting is embedded). Both settings rely upon information that is derived from non-quantitative sources; both settings demand room for negotiation and space for political contestation rather than just technical resolution in decision-making.

A collection of papers prepared for an International Conference on Operational Research for Development held in Ahmedabad in 1992 [18] offered a broad range of applications across the fields of agriculture, water and energy, health, transportation and distribution, and business applications. Prior conferences of the Operational Research Society had regularly included themes on Operational Research *in* Developing Countries, but the Ahmedabad Conference on Operational Research *for* Development and the various initiatives that flowed from it represented an explicit shift in acknowledging the two-way movement in ideas and applications between the developed and the developing world.

This two-way flow and the underpinning provided by new paradigm techniques to both fields of Community Operational Research and Operational Research for Development are exemplified by the work of Naman and colleagues [19,20]. They report on the use of problem structuring methods by a small community in Brazil to explore alternatives for the improvement of life in such poor communities through enhanced self-management and sustainability in food production.

### Planning public health systems interventions

As had been demonstrated by the work of the Foresight Committee on obesity [8], the task of developing a whole systems understanding of the causal mechanisms that lie behind a major public health issue can seem relatively trivial when measured in relation to the task of deriving meaningful interventions designed to tackle those issues. The more the complexity of our understanding the harder is the task of identifying points and mechanisms of intervention.

Having (often painstakingly) built up an understanding of the complexities, the identification of meaningful interventions will invariably necessitate a step back towards simplification – de-complexifying the complexities to sort out critical factors and critical interactions between them. Complexity is frequently most appropriately portrayed in the form of a visual map, and an initial stage in most problem structuring approaches is to assist in the construction of visual maps. These may be based on a review of the authoritative literature (where a broad overarching policy is being formulated) or upon the solicited views of key informants (where a more finely-tuned localised strategy is needed).

The Foresight Committee employed causal loop modelling, explained in detail by Vandenbroek, Goossens and Clemens [21], which produced individually and then fused together a series of individual maps contained within a composite atlas [22]. The Foresight Committee employed the composite map to highlight “how agents outside conventional mechanisms are key enablers and barriers to change”. The Committee Report [8] incorporated a simplified map which clustered areas thematically and identified critical spheres of influence and the most important actors within them.

Drawing this approach into routine public health systems planning presents new challenges. The amount of time and resources and the degree of highly specialised technical support deployed by the Foresight Committee will only be available for a ‘one-off’ exercise conducted at the highest level. Implementing a Primary Health Care Approach with its emphasis on community and locality involvement requires a more replicable approach – though it should be recognised that the replication itself will allow for more technical sophistication and more complex portrayals to accrue over time. However, in the first instance, the more limited time and resources available to strategic health planners practising in developing countries will necessitate use of a more rapid process.

**Figure 1:**
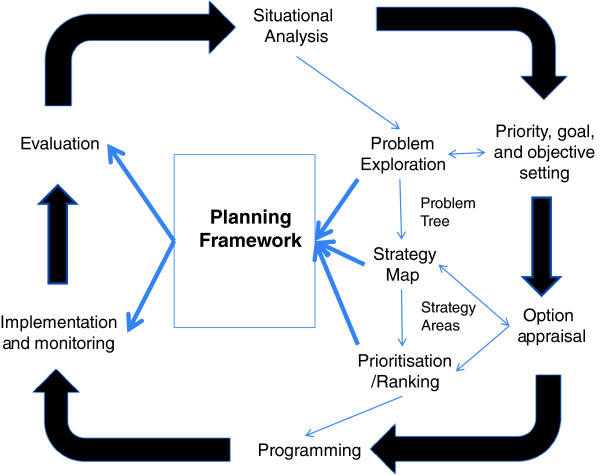
Problem structuring within a planning spiral.

Thunhurst and Barker [23] present an approach, initially developed for district level health planning in Pakistan and subsequently incorporated into the District Implementation Planning process in Malawi. {Both initiatives were undertaken in collaboration with the respective Ministries of Health – the former under an ADB/ODA funded project which provided technical assistance from the Nuffield Institute for Health at the University of Leeds; the latter under an EU funded project}. This guides district level health planners through an initial problem exploration process to the preparation of a strategy map on the basis of which a prioritisation exercise can be undertaken. This approach built upon a planning framework (derivative of the logical framework) designed to draw out the different planning horizons over which decision-making has to be framed. It employed a problem tree to differentiate underlying causes from their more immediate manifestations and thus to isolate core problems which were then mapped into strategy areas. These were prioritised employing a ranking exercise. Throughout the processes were explicitly designed to be participative, prompting involvement from community representatives and from other sectors crucial to the resolution of the underlying core problems. Figure 1 shows the positioning of these stages of analysis within a conventional planning spiral. The resultant planning framework and the respective districts’ strategic maps captured the more participative and more considered analysis and provided a more realistic assessment of what could be achieved within short and medium term planning horizons.

**Figure 2:**
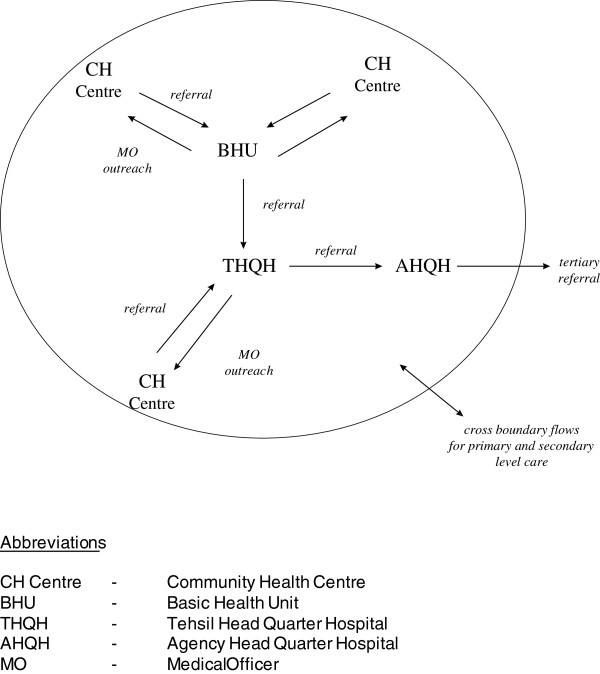
The agency based health care delivery system.

A similar approach was used to develop a *Master Health Plan* for the Federally Administered Tribal Areas of Pakistan [24]. {This planning exercise was undertaken in collaboration with a team from the Ministry of Health for the NWFP Province and was managed by the British Council under DFID-funded components of the Family Health Project}. This Master Plan was aimed at turning a previously fragmented health care delivery system into a coherent Agency-Based Health-Care Delivery System {Figure 2} along the lines of the District Health Model being promoted by the WHO. To formulate this new system a problem analysis exercise was conducted drawing upon the Strategic Choice Approach [25], one of the new paradigm PSMs. Sadly, shortly following the formulation of the newly designed system, the events of September 2001 threw that area of the world and particularly that area of Pakistan into turmoil, prohibiting further implementation.

Although these applications of PSMs had encouraged and assumed a high degree of community involvement in health care planning, the most explicit use of these approaches to draw community organisations into statutory planning processes was made in the Republic of Ireland. Thunhurst, Cronin & Curtin [26] report on the work of the ORCHID (Operational Research for Community Health Institutional Development) Project based at University College Cork conducted with the Northside Initiative for Community Health (NICHE) community health development project which covers a deprived area of north Cork^a^. (It was no coincidence that all three members of the ORCHID Project had extensive experience of working within developing countries, the importance of which they readily acknowledged). The planning exercises undertaken with NICHE also employed an adaptation of the Strategic Choice Approach problem structuring method [25] and built upon the findings of a participatory planning exercise previously carried out by the NICHE Project. The broad range of issues identified within this prior exercise (which covered both immediate health care delivery issues and underlying determinants of health) were prioritised and incorporated into a Strategic Health Plan for the NICHE Project which formed the basis for their subsequent negotiation with statutory bodies. This prompted a distinct attitudinal change within the statutory bodies who had previously regarded local planning as a technical matter beyond the competence of community representatives. It also contributed to an acceleration in the improvement of general practice facilities within the area.

As well as supporting decentralised planning processes new paradigm problem structuring methods can be used to assist conflict resolution – or conflict amelioration. Thunhurst [27] reports the use of cognitive mapping (on which the SODA methodology is based) and illustrates how political mapping can be employed to clarify and represent the views and interests of respective antagonists. In this instance these approaches were adopted in a polarised debate concerning the introduction of waste incineration in Ireland. They helped to reveal areas of agreement as well as clarifying the precise nature of the disagreements between the respective parties.

## Discussion

The Millennium Development Goals relating to health gain set ambitious objectives for developing health sectors as did their predecessors, the targets formulated for the achievement of Health for All by the year 2000. The failure to achieve these targets had multiple causes. Although formulated at a time that provided adequate lead time, their wider adoption generally occurred at too late a stage to make them any longer achievable. Even if earlier adoption had been achieved it is likely that they would have proved over-ambitious. What they did do was to highlight the importance of strengthening strategic planning systems – planning systems which link and enable translation of higher order aspirations into short-term operational plans. They also highlighted the need for a broader systems vision, one which could convert the aspirations of the Primary Health Care Approach, community and inter-sectoral engagement, into a reality.

The subsequent adoption of a whole systems perspective which views health systems as a set of discrete but interlinked sub-systems has been embedded within the WHO’s “building blocks approach” [28]. This encourages countries to adopt a framework of understanding based upon analysis of six sub-systems: health service delivery, health workforce, health information systems, access to essential medicines, health systems financing and leadership and governance. This framework has now been widely adopted within developing countries to structure strategic plans at a national and local level – see, for example, the recently prepared Health Sector Strategic Plan 2012–16 for the Republic of Sudan [29].

Thus, it could be argued that the embedding of the whole systems perspective into the long term plans of developing health systems has been largely achieved. However, the ability to undertake appropriate analysis to support this thinking has (inevitably) lagged behind, as has the integration of planning systems that enable translation into whole systems action. It is not argued here that problem structuring methods provide a panacea. They can provide a valuable weapon in the technical armoury of whole systems planning, as can more established methods such as rapid appraisal. Above all, as we believe that the illustrations presented above demonstrate, they can enrich planning processes to ensure that the wider whole systems perspective is retained when planning is carried down to the micro (district/community) level.

The most discernible impact of the enrichment of health systems development will be in the strengthening that results in strategic planning systems. Not only will this be more analytic and more ‘joined-up’, in that the longer term implications of short-term decisions will be addressed, but it will also be more participative, in that the wider information base drawn upon and the need for broader interpretation, will predicate community involvement in the subsequent stages of the planning spiral. Some specific actions to enhance this process are given below.

Developed countries have as much to learn from this as do their developing counterparts. Over recent years the WHO has worked with international donors and Ministries of Health in a number of developing countries to form the International Health Partnership (IHP). Under the auspices of the International Health Partnership Plus programme and employing guidance developed within that programme countries undertake a Joint Assessment of National Health Strategies and Plans (JANS) [30] whereby international expertise and international experience is drawn upon to ensure that national health strategies and plans meet agreed norms (specified in IHP+ guidance documents). The purpose (as yet to be fully tested) is to eliminate the time consuming and repetitive regularity with which ministries are required to prepare, ab initio, complex documentation to meet the distinct requirements of each individual putative health sector donor. On a recent JANS mission to Sudan the visiting team (of which this author was a member) was asked why it was that developed countries themselves did not undertake a JANS process. The reality is that the real-politic of global health sector funding means that developed countries are not required to undergo as rigorous a process of scrutiny as are developing countries. The truth however is also that if they were so to subject themselves to such a scrutiny process it would undoubtedly find them wanting. Specifically, the paucity and the fragmentation of planning processes involving both the health sector and the complementary sectors whose plans and interventions are critical to the production of good or bad health would be viewed with some derision. And the involvement of communities within these processes would be seen as tokenistic at best. Sectoral planning in developed countries remains heavily silo-based; a dearth in inter-sectoral public health planning is the inevitable consequence. The luxury of the relative level of resources available to developed countries is that such major irrationalities can go unchecked and largely unobserved. This should not however allow it to go unrecognised that the development of whole systems public health planning processes with fuller community involvement could (in aggregate) offer even greater benefits, in terms of the overall utilisation of health sector resources, to developed countries than they would to developing countries.

Achieving parallel paradigmatic shifts – in our understanding of the nature of public health systems and in our use of newly emerging analytic techniques to further that understanding – is no mean task. It is one which calls for a globalised effort – a recognition that no part of the world has a monopoly on insights and experiences. The developed world may have the edge on developing nations in their deployment of more sophisticated analytic techniques; but it is the developing nations that can instruct their more developed counterparts on the achievement of community engagement and of the practice of inter-sectoral involvement.

## Conclusions

To realise the full health gain achievable from translating the paradigmatic shifts from the conceptual to the practical level requires action across all levels of public health systems. In particular:

1. At the macro (mainly national) level, it is essential to maintain the integrity of a whole systems approach resisting the further proliferation of separate disease-specific programmes and interventions and to establish a planning framework which guards against fragmentation at lower levels within the health system.

2. At the meso (mainly regional) and micro (mainly local) levels, it is essential to ensure the active involvement of community representatives and of representatives from parallel sectors for whom health outcomes are secondary to their principal objectives. This involvement should be built in at a sufficiently early stage that it is not merely reactive but recognises the formative importance of their autonomous and of their mutual interventions.

3. At all levels, it is essential to provide access to appropriate analytic skills. Given the global pressures on human resource budgets this is unlikely to take the form of the development of new cadres of public health systems analysts but of expanding the remit of established cadres. In particular, public health planners should be empowered to adopt a whole systems perspective rather than simply being short term manipulators of inherited levels of relatively fixed resources. Statistical officers should be trained to extend their skills beyond the analysis of quantitative data generated within the health sector to the collection and the analysis of data (which will frequently be of a qualitative nature) from across the full range of health determining actors and activities.

The most distinctive feature of the confluence between the River Kabul and the River Indus is the speed with which the two very distinguishable currents merge. Within a few hundred metres the River Indus has absorbed the distinct characteristic of the River Kabul into its generally stronger flow. In this paper we have looked at the coming together of a number of similarly previously distinct currents – the currents of whole systems thinking and of whole systems analysis, and the respective currents of health sector development in developed and developing countries. Although we would strongly discourage the search for universal solutions to issues of health care delivery (which have historically led to an inappropriate importation of solutions from the developed world by the developing world), we would argue and urge for more synergy and sharing of thinking. It is the much-needed confluence in our understanding of public health care systems, with developed countries adopting the more holistic vision of public health planning that is demanded of developing countries, which should provide the framework for the confluence in accompanying forms of analysis.

## Endnote

^a^Ironically, it is the area of north Cork (Knocknaheeny) which has recently acquired some international notoriety as standing adjacent to the offices through which the Apple Computers corporation routes its European undertakings for maximum tax advantage.

## Abbreviations

PHC: Primary health care; OR: Operational research; SA: Systems analysis; MS: Management sciences; PSMs: Problem structuring methods; NICE: National institute for health and clinical excellence; SODA: Strategic options and development analysis; CELAT: Centro Latinamericano do Trabajo social; WHO: World Health Organisation; NICHE: Norrthside initiative for community health; ORCHID: Operational research for community health institutional development; JANS: Joint assessment of national health strategies and plans; IHP: International health partnership.

## Competing interests

The author is a semi-retired independent consultant who works in developed and developing countries on a range of projects involving the analysis of public health systems.

## Author’s information

The author holds first and second degrees in the fields of statistics and operational research, subjects in which he held academic positions for twenty years. Through his subsequent appointment to a post at the Nuffield Institute for Health at the University of Leeds he made a gradual transition into health planning and subsequently into public health. His doctorate was in the use of problem structuring methods to enhance public health discourse. The author worked extensively in developing countries, for a period of four years in Pakistan and for a period of just over a year in Nepal, as project director for health systems strengthening projects. He has latterly held posts within the Department of Epidemiology and Public Health at University College Cork and within the Faculty of Health and Life Sciences at Coventry University and has conducted numerous short-term consultancies in Asia, Africa and the Middle East.
